# The mechanism linking self-determined motivation to pickleball continuance intention: mediating role of leisure specialization and gender heterogeneity

**DOI:** 10.3389/fpsyg.2026.1806235

**Published:** 2026-09-15

**Authors:** Yajun Wu, Zhidong Dai, Soohyun Kim, Xin Lyu, Kailin Zhong

**Affiliations:** 1 School of Physical Education, Chuzhou University, Anhui, China; 2 Department of Sport and Healthcare, Namseoul University, Cheonan, Republic of Korea; 3 School of Physical Education and Sports, Soochow University, Jiangsu, China; 4 Department of Sport Convergence, Namseoul University, Cheonan, Republic of Korea

**Keywords:** continuance intention, gender heterogeneity, leisure specialization, mediating effect, pickleball, self-determined motivation

## Abstract

Integrating Self-Determination Theory (SDT) and the Theory of Planned Behavior (TPB), this study examines how self-determined motivation (autonomy, competence, relatedness) shapes continuance intention among pickleball participants, with leisure specialization conceptualized as a domain-specific operationalization of perceived behavioral control (PBC). The mediating role of leisure specialization and the moderating effect of gender on the underlying motivational pathways are tested. Drawing on cross-sectional data from 789 Chinese tournament participants, structural equation modeling (SEM) reveals that all three dimensions of self-determined motivation exert significant positive effects on leisure specialization, which in turn partially mediates the relationship between self-determined motivation and continuance intention. Gender, however, does not significantly moderate the pathways linking motivational dimensions to leisure specialization, suggesting that the core motivational mechanism operates comparably across male and female participants. The findings provide empirical support for the pathway of “psychological need satisfaction → behavioral control (leisure specialization) → sustained participation,” and suggest that pickleball's low-skill-threshold design and mixed-gender format may help attenuate traditional gender-role barriers to sport participation. By validating this context-specific operationalization of PBC, the study extends integrated SDT-TPB research and offers practical insights for promoting sustained participation in emerging low-threshold sports.

## 1 Introduction

Pickleball, an emerging racquet sport combining competitive intensity with social interaction, has expanded rapidly from a family pastime into a global phenomenon, driven by its low skill threshold, lightweight equipment, and simple rules (Leach and Colgate, 1982). Global participation surpassed 50 million in 2023 (International Federation of Pickleball, 2023), yet this growth conceals a persistent challenge: converting first-time trialists into long-term, committed participants. Unlike traditional sports with established participation cultures, emerging activities depend heavily on retaining individuals beyond the initial novelty phase (Wang et al., 2023). In China, where pickleball has gained remarkable traction in recent years, participant profiles differ notably from the older-adult samples commonly reported in Western studies—a demographic distinction that raises important questions about whether existing motivational models, developed largely in Western contexts, adequately explain sustained participation among young and middle-aged Chinese adults.

Central to addressing this challenge is the construct of continuance intention, defined as an individual's volitional commitment to persist in an activity across future occasions (Zeithaml et al., 1996). Within behavioral science, it is widely recognized as the strongest proximal predictor of long-term behavioral maintenance, serving as the critical bridge between motivational states and sustained action (Li et al., 2025). Unlike initial participation willingness, this construct reflects a resolved cognitive-motivational state shown to mediate the well-documented “intention-behavior gap” (Ajzen, 1991). Empirically, studies across various sport settings confirm that continuance intention strongly predicts actual re-participation and exercise persistence (Choi, 2023; Karakullukcu et al., 2025). Theoretically, it constitutes the ultimate dependent variable of interest for understanding not only why individuals initiate an activity, but why they remain engaged.

The practical significance of continuance intention is especially pronounced for emerging sports such as pickleball. For event organizers, facility operators, and sport promotion agencies, the long-term viability of a new sport hinges not on one-off tournament attendance but on cultivating a stable, recurring participant base capable of generating sustained economic and social value (Wang et al., 2023). This pattern parallels evidence from China's ice-and-snow industry, where the transition from “experiential engagement” to “habitual participation” has been identified as the core bottleneck for sustainable development (Liang et al., 2025). Without a clear understanding of continuance intention drivers, promotional efforts risk prioritizing short-term buzz over the relational, skill-based, and identity processes that foster enduring involvement—a risk particularly acute for pickleball, given its rapid but fragile growth in China.

Despite its importance, the motivational mechanisms underlying pickleball continuance intention remain largely unexplored, particularly within the Chinese young-adult context. Prior research on pickleball has predominantly examined older Western samples, emphasizing health benefits, social connectedness, and leisure satisfaction (Heo et al., 2018; Casper and Jeon, 2018). It remains unclear whether the same factors operate among Chinese participants who are younger, more achievement-oriented, and embedded in distinct cultural and time-constrained environments. Moreover, although both Self-Determination Theory (SDT) and Theory of Planned Behavior (TPB) have been individually applied to explain sport participation, existing integrated SDT-TPB studies generally treat perceived behavioral control (PBC) as a general perceptual construct, lacking domain-specific and behavioral operational indicators in leisure contexts. Empirically, existing pickleball research in China has been largely confined to descriptive analyses of participation status, event operation, and industry promotion (Wang et al., 2023), with no published studies examining the psychological mechanisms driving long-term participation. This leaves the motivational mechanism underlying sustained participation among young and middle-aged adults an open empirical question. In particular, the integrated application of the two theories—especially through the lens of leisure specialization as a domain-specific operationalization of perceived behavioral control—has yet to be tested in pickleball or similar low-threshold, socially oriented sports.

This study addresses this gap by proposing an integrated model that positions leisure specialization—a multidimensional construct encompassing behavioral involvement, cognitive mastery, and affective commitment (Scott and Shafer, 2001)—as the mediating mechanism through which self-determined motivation (autonomy, competence, relatedness) influences continuance intention. The theoretical rationale for this proposition is systematically presented in Table 1, which aligns the three core dimensions of PBC in TPB (Ajzen, 1991)—namely, behavioral execution control (resource dominance), perceived capability (ease/difficulty assessment), and affective commitment value (Pelletier et al., 1995)—with the three corresponding dimensions of leisure specialization (Scott and Shafer, 2001): behavioral involvement (time/money/equipment investment), cognitive mastery (skill/knowledge level), and affective commitment (meaning/emotional attachment).

**Table 1:** Theoretical alignment of leisure specialization as an operationalization of PBC.

Core connotation of PBC (Ajzen, 1991)	Leisure specialization dimension (Scott and Shafer, 2001)	Alignment mechanism	Empirical support in pickleball
Behavioral execution control (resource dominance)	Behavioral involvement (time/money/equipment investment)	Time, financial and equipment investment reflects perceived control over participation resources	High-investment group (≥3 sessions/week): 39.8% (Table 2); behavioral involvement parcel loading = 0.754 (Table 3)
Perceived capability (ease/difficulty assessment)	Cognitive mastery (skill/knowledge level)	Specialized cognition reinforces efficacy beliefs for sustained participation	Competence → Specialization path β = 0.285 (Table 7); COM6 (skill confidence) loading = 0.843 (Table 3)
Affective commitment value (volitional control, Pelletier et al., 1995)	Affective commitment (meaning/emotional attachment)	Emotional attachment indirectly consolidates perceived behavioral control	Relatedness mediation proportion via affect: 20.13% (Table 8); affective commitment parcel loading = 0.778 (Table 3)

This theoretical alignment is grounded in established leisure literature. First, behavioral involvement reflects resource investment and behavioral proficiency, corresponding to the resource-based dimension of PBC; individuals who invest greater time and resources in an activity tend to develop greater behavioral mastery and perceived control over their participation (Scott and Shafer, 2001; Havitz and Mannell, 2005). Second, cognitive mastery captures the accumulation of knowledge and skills, thereby reinforcing efficacy beliefs relevant to continued participation; as participants acquire deeper activity-specific knowledge, their confidence in navigating participation challenges increases (Bryan, 2000; Lee and Scott, 2004). Third, affective commitment reflects emotional attachment to the activity, which may further consolidate perceived control through internalization processes (Pelletier et al., 1995); as an activity acquires personal meaning, participants perceive greater ease and willingness to sustain involvement (Kyle et al., 2007; Stebbins, 2007).

**Table 2:** Participant demographic characteristics (*N* = 789).

Characteristic	Category	Frequency	Percentage (%)
Gender	Male	507	64.26
	Female	282	35.74
Age	10–19 years	23	2.92
	20–29 years	255	32.32
	30–39 years	230	29.15
	40–49 years	188	23.83
	50–59 years	64	8.11
	≥60 years	29	3.68
Education level	Junior high school	28	3.55
	High school	56	7.10
	Associate degree	226	28.64
	Bachelor's degree	305	38.66
	Graduate degree	174	22.05
Pickleball participation duration	< 6 months	39	4.94
	6 months to < 1 year	135	17.11
	1–2 years	255	32.32
	2–3 years	189	23.95
	3–5 years	98	12.42
	>5 years	73	9.25
Weekly participation frequency	≤ 1 session	63	7.98
	2–3 sessions	412	52.22
	>3 sessions	314	39.80
Daily participation duration	≤ 1 h	261	33.08
	2–3 h	384	48.67
	>3 h	144	18.25

**Table 3:** Confirmatory factor analysis (CFA) results (*N* = 789).

Construct	Item	Unstd. loading	**S.E**.	**C.R**.	***p***	Std. loading	AVE	CR
Relatedness	REL1	1.000	–	–	–	0.841	0.618	0.890
	REL2	0.806	0.032	24.818	^***^	0.773		
	REL3	0.797	0.033	24.144	^***^	0.758		
	REL4	0.835	0.033	25.609	^***^	0.790		
	REL5	0.785	0.032	24.481	^***^	0.766		
Competence	COM6	1.000	–	–	–	0.843	0.615	0.889
	COM7	0.801	0.031	25.530	^***^	0.787		
	COM8	0.797	0.031	25.323	^***^	0.782		
	COM9	0.762	0.031	24.432	^***^	0.763		
	COM10	0.749	0.032	23.596	^***^	0.744		
Autonomy	FRE11	1.000	–	–	–	0.803	0.588	0.877
	FRE12	0.848	0.037	22.859	^***^	0.762		
	FRE13	0.825	0.037	22.396	^***^	0.750		
	FRE14	0.863	0.037	23.415	^***^	0.777		
	FRE15	0.788	0.036	21.995	^***^	0.739		
Leisure specialization	COG	1.000	–	–	–	0.746	0.577	0.803
	ACT	1.052	0.053	19.976	^***^	0.754		
	EMO	1.002	0.049	20.586	^***^	0.778		
Continuance intention	MPI1	1.000	–	–	–	0.864	0.690	0.899
	MPI2	0.939	0.031	30.687	^***^	0.856		
	MPI3	0.746	0.028	26.270	^***^	0.777		
	MPI4	0.866	0.030	28.670	^***^	0.821		

^*^p < 0.05, ^**^p < 0.01, ^***^p < 0.001. S.E. was estimated via the Bootstrap method; CR, composite reliability; AVE, average variance extracted.

This conceptualization is particularly suitable for pickleball, given its characteristics as a form of “low-threshold serious leisure” (Stebbins, 1982). Specifically, pickleball's low skill threshold facilitates rapid performance feedback—beginners can reach a competitive level in approximately one-third of the time required in tennis (International Federation of Pickleball, 2023)—and its mixed-gender formats, which characterize 87% of activities (Casper and Jeon, 2018), may foster emotional bonds and internalization of participation motives, thereby providing a more nuanced framework for assessing PBC through the lens of leisure specialization.

This integration is not an *a priori* theoretical exercise; rather, it emerges directly from the research problem at hand. Explaining why young Chinese pickleball players sustain their involvement requires a framework that simultaneously accounts for the motivational quality of the experience (SDT) and the perceived capacity to continue participating (TPB). Leisure specialization, as a context-specific indicator of perceived behavioral control, provides the theoretical bridge connecting these two well-established yet previously disconnected perspectives. Gender differences in the motivational pathways are also explored as a supplementary analysis.

In sum, this study aims to empirically verify the mediating pathway of “self-determined motivation → leisure specialization (as perceived behavioral control) → continuance intention” among Chinese pickleball tournament participants, and to explore the potential moderating role of gender within this pathway.

## 2 Literature review

### 2.1 Self-determination theory in sports participation

Self-Determination Theory (SDT; Ryan and Deci, 2000; Deci and Ryan, 2000) constitutes one of the most influential frameworks for understanding motivation in sport and physical activity contexts. The theory posits that satisfaction of three basic psychological needs—autonomy, competence, and relatedness—is essential for fostering intrinsic motivation and sustaining behavioral engagement (Ryan and Deci, 2000). Autonomy refers to the experience of volition and choice in one's actions; competence involves perceptions of effectiveness and mastery; and relatedness concerns the experience of meaningful connection with others.

A substantial body of empirical research has supported the applicability of SDT across diverse sport settings. In competitive sport, Pelletier et al. (1995) demonstrated that need satisfaction predicts athletes' self-determined motivation, which in turn influences performance and persistence. In recreational sport, studies have similarly shown that need satisfaction enhances enjoyment, commitment, and continued participation (Wilson and Rodgers, 2004; Hagger et al., 2006). However, most SDT research in sport has focused on traditional competitive activities with well-established participation cultures. The extent to which SDT applies to emerging recreational sports characterized by low skill thresholds and mixed-gender participation formats remains underexplored.

Recent SDT research has extended these findings to diverse sport settings. Ntoumanis and Moller (2025) provided a comprehensive review of SDT's application to physical activity, reaffirming that need satisfaction is fundamental to sustained behavioral engagement. In elite volleyball contexts, Morbée et al. (2024) demonstrated that need-supportive coaching styles significantly predicted athletes' continued participation, whereas need-thwarting styles undermined persistence. Haberstock et al. (2024) further showed that peer-created climates significantly influence basic psychological need satisfaction, underscoring the importance of social environments beyond coach-athlete interactions.

### 2.2 Theory of planned behavior and perceived behavioral control

The Theory of Planned Behavior (TPB; Ajzen, 1991) offers a complementary perspective on behavioral persistence by emphasizing perceived behavioral control (PBC) as a key determinant of intention. PBC refers to individuals' perceptions of their resources, capabilities, and opportunities to perform a given behavior (Ajzen, 1991). Within sport participation contexts, PBC encompasses such factors as confidence in one's skills, access to necessary resources, and perceived ease of participation (Hagger et al., 2006).

TPB has been widely used to explain participation intentions and behavior in sport. Prior research has consistently shown that PBC significantly predicts both intention and actual participation across a range of sport settings (Hagger et al., 2006). Nevertheless, the conceptualization and measurement of PBC in this literature have generally relied on broad, general perceptions of control rather than domain-specific indicators. To address this limitation, the present study conceptualizes leisure specialization—a multidimensional construct encompassing behavioral involvement, cognitive mastery, and affective commitment—as a domain-specific operationalization of PBC.

Recent applications of TPB to sport participation have further supported its predictive utility. Hopkins and Hopkins (2026) found that attitudes and subjective norms significantly predicted adolescent female athletes' intentions to continue sport participation. Additionally, Ebrahimi et al. (2022) demonstrated that within-person variations in PBC predicted subsequent sedentary leisure time, underscoring the value of capturing behavioral control at the situational level.

### 2.3 Leisure specialization as behavioral control

Scott and Shafer's (2001) leisure specialization framework offers a comprehensive approach to understanding behavioral engagement in leisure activities. Within this framework, specialization is conceptualized as a multidimensional construct comprising three dimensions: behavioral involvement (time, effort, and resource investment), cognitive mastery (knowledge and skill development), and affective commitment (emotional attachment and activity identity).

Conceptually, PBC in TPB comprises two related components: perceived self-efficacy (judgments of capability to perform the behavior, including skill mastery and ease of execution) and perceived controllability (judgments of volitional control, including resource access and willpower; Ajzen, 2002). Control beliefs underlying PBC encompass both internal factors (skills, knowledge, volition) and external factors (time, finances, social support), which jointly determine perceived capacity to sustain participation.

This two-dimensional, three-layer conceptualization of PBC aligns closely with the three dimensions of leisure specialization. First, behavioral involvement reflects time, financial and equipment investment, corresponding to the resource control layer of PBC; greater resource input directly enhances individuals' perceived controllability over participation. Second, cognitive mastery captures the accumulation of activity-specific knowledge and skills, corresponding to the perceived self-efficacy component of PBC; deeper skill mastery strengthens efficacy beliefs for sustained participation. Third, affective commitment reflects emotional attachment and identity integration with the activity, corresponding to the volitional-control layer of PBC; internalized emotional meaning further reinforces perceived control through motivational internalization processes (Pelletier et al., 1995).

This operationalization responds to the call by Hagger et al. (2006) for more domain-specific and behavior-grounded assessments of PBC, moving beyond general perceptual measures to capture concrete behavioral manifestations of control in recreational sport settings. Taken together, this alignment suggests that leisure specialization can serve as a context-specific operationalization of PBC, particularly in low-threshold sport settings.

Recent empirical studies have provided robust support for this framework. Tian et al. (2022) found that leisure specialization dimensions (behavior, cognition, affect) positively predicted runners' psychological commitment and life satisfaction. Liang et al. (2025) demonstrated that leisure motivation influences leisure specialization through negotiation strategies, establishing a pathway from motivational antecedents to deep engagement. Huo et al. (2025) further confirmed that leisure specialization enhances leisure satisfaction through place dependence and place identity, suggesting that specialization serves as a behavioral manifestation of internalized psychological needs.

### 2.4 Gender differences in sports motivation

Gender differences in sport motivation have been extensively examined, with traditional gender role perspectives proposing that males and females may hold distinct motivational orientations: males are more likely to prioritize competence demonstration and skill validation, whereas females tend to place greater emphasis on social support and relatedness (Chalabaev et al., 2013; Kipp and Weiss, 2013).

Recent studies, however, have increasingly questioned the universality of such gender differences. Manzano-Sánchez (2023), examining motivational profiles among 752 students, found no significant gender differences in the relationship between self-determined motivation and intention to be physically active. Similarly, Li et al. (2025) reported that although gender moderated certain peripheral pathways in an integrated BPN-TPB model for adolescent exercise persistence, the core motivational mechanisms remained largely invariant across genders. Collectively, these findings suggest that sport-specific characteristics may shape or attenuate gendered motivational patterns: sports with low skill thresholds and mixed-gender participation formats may reduce traditional gender disparities by lowering barriers to entry and fostering more inclusive social environments (Cerezuela et al., 2023).

Pickleball, with its simplified rules, lightweight equipment, and predominantly mixed-gender formats −87% of activities involve mixed doubles (Casper and Jeon, 2018)—provides an appropriate context for examining whether such low-threshold sport characteristics moderate gender differences in the proposed motivational pathways.

### 2.5 Integrated application of SDT and TPB in sport persistence

Integrating SDT and TPB has gained traction as a framework for explaining sustained behavioral engagement. Gucciardi and Jackson (2015) demonstrated that basic psychological need satisfaction predicted TPB antecedents (attitudes, PBC, subjective norms), which in turn predicted sport continuation. Li et al. (2025) extended this integration to adolescent exercise persistence, confirming a sequential pathway from perceived autonomy support to basic psychological need satisfaction, through TPB constructs, to behavioral intention and persistence. A key innovation within this integrated stream lies in operationalization of perceived behavioral control through domain-specific indicators. Rather than relying on general perceptions of control, leisure specialization—encompassing behavioral involvement, cognitive mastery, and affective commitment (Scott and Shafer, 2001)—provides a contextually grounded measure of PBC in recreational sport settings, responding to calls for more nuanced assessments that capture actual behavioral manifestations of control (Hagger et al., 2006).

## 3 Research framework and hypotheses

### 3.1 Research framework

Drawing on Self-Determination Theory (SDT; Ryan and Deci, 2000) and the Theory of Planned Behavior (TPB; Ajzen, 1991), this study develops the conceptual model presented in Figure 1. The model proposes that the three dimensions of self-determined motivation (autonomy, competence, relatedness) exert positive effects on continuance intention through the mediating role of leisure specialization, with gender moderating the relationships between the motivational dimensions and leisure specialization. Rather than aggregating the three needs into a single latent construct, this study treats them as distinct exogenous variables in order to examine their differentiated effects on leisure specialization and to identify the relative importance of each motivational dimension. Within this framework, leisure specialization is conceptualized as a domain-specific operationalization of PBC, and serves as a critical mediating mechanism through which psychological needs are translated into sustained behavioral intention.

**Figure 1:**
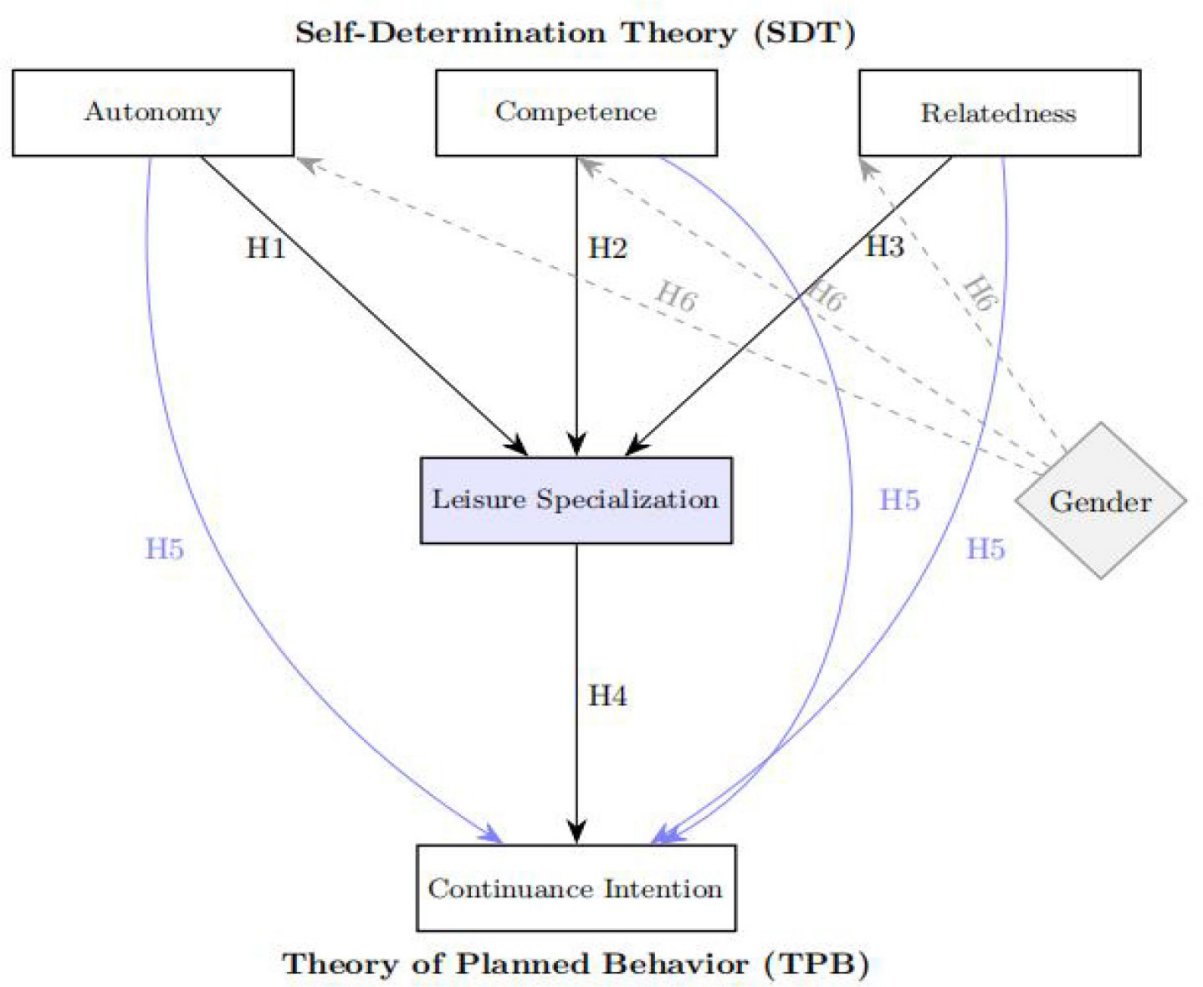
The influence of self-determined motivation on continuance intention.

### 3.2 Hypothesis development

Based on the theoretical integration established in the preceding literature review (Sections 2.1–2.5), the following hypotheses are formulated.

Self-Determination Theory posits that satisfaction of the three basic psychological needs—autonomy, competence, and relatedness—facilitates intrinsic motivation and deeper behavioral engagement (Ryan and Deci, 2000). Within leisure contexts, need satisfaction has been linked to sustained involvement and activity-specific investment (Ryan and Deci, 2000; Tang et al., 2021; Gui et al., 2018). Given pickleball's accessible design and varied participation formats, which afford opportunities for volitional choice (autonomy), rapid skill feedback and early mastery experiences (competence; Ryu et al., 2020), and social interaction (relatedness)—each need dimension is expected to positively predict leisure specialization.

The Theory of Planned Behavior identifies perceived behavioral control (PBC) as a proximal antecedent of behavioral intention (Ajzen, 1991). As argued in Section 2.3 and Table 1, leisure specialization—comprising behavioral involvement, cognitive mastery, and affective commitment—serves as a domain-specific operationalization of PBC within recreational sport settings. Empirical research demonstrates that specialization predicts sustained engagement and commitment (Liang et al., 2025; Tian et al., 2022; Huo et al., 2025). Consistent with this logic, leisure specialization is expected to positively predict continuance intention.

Integrating the SDT-TPB framework suggests that psychological needs translate into behavioral intention via behavioral control mechanisms (Vallerand, 1997; Li et al., 2025). Specifically, need satisfaction fosters resource investment, skill confidence, and emotional attachment—dimensions captured by leisure specialization—which in turn strengthen perceived behavioral control and the willingness to sustain participation. Leisure specialization is thus expected to act as a mediating mechanism in these relationships.

Regarding gender, traditional role theories suggest the possibility of differences in motivational orientations (Chalabaev et al., 2013), although recent evidence from low-threshold sports questions the universality of such differences (Manzano-Sánchez, 2023; Li et al., 2025). Pickleball's mixed-gender format and low skill threshold may further attenuate traditional gender-based barriers (Cerezuela et al., 2023). This provides a basis for testing potential gender differences in the strength of these motivational paths.

Based on the above theoretical reasoning and empirical evidence, the following formal hypotheses are proposed:

- **H1:** Autonomy need exerts a significant positive effect on leisure specialization among pickleball participants.
- **H2:** Competence need exerts a significant positive effect on leisure specialization among pickleball participants.
- **H3:** Relatedness need exerts a significant positive effect on leisure specialization among pickleball participants.
- **H4:** Leisure specialization exerts a significant positive effect on continuance intention among pickleball participants.
- **H5:** Leisure specialization partially mediates the positive relationships between each of the three self-determined motivation dimensions (autonomy, competence, relatedness) and continuance intention.
- **H6:** Gender moderates the positive relationships between the three self-determined motivation dimensions and leisure specialization, such that path strengths differ across male and female participants.

## 4 Methods

### 4.1 Participants and procedure

This study employed purposive sampling to recruit participants from the 2024 Chinese National Pickleball Open, regional tour events, and University Championships. A pilot study (*N* = 30) was conducted 4 weeks prior to the main survey to refine item clarity and assess potential response bias. Ethical approval was obtained from the Ethics Committee of Chuzhou University (Approval No. CZSC2026-001).

Electronic questionnaires were distributed via QR codes disseminated through official tournament communication channels, with on-site researchers guiding participants to complete the survey during intervals between matches. A total of 802 questionnaires were distributed. After excluding 13 invalid responses, 789 valid responses were retained, yielding an effective response rate of 98.4%. The high response rate is attributable to on-site distribution and official tournament endorsement, both of which fostered strong participant cooperation and willingness to complete the survey.

The final sample comprised 507 males (64.26%) and 282 females (35.74%), with 85.3% of participants aged between 20 and 49 years. Detailed demographic characteristics (including age, education level, participation duration, weekly participation frequency, and daily participation duration) are presented in Table 2. As participants were purposively sampled from tournament settings, the sample predominantly represents competitive, high-involvement pickleball players; findings may therefore not generalize fully to recreational or novice player groups.

### 4.2 Measures

All scales were originally developed in English. Following standard cross-cultural adaptation procedures, we used back-translation: two bilingual sport psychology researchers independently translated items into Chinese, and two others back-translated the Chinese version into English. Discrepancies were resolved via group discussion, and wording was refined based on pilot feedback to ensure semantic equivalence. Unless otherwise noted, all items used a five-point Likert scale (1 = “Strongly Disagree” to 5 = “Strongly Agree”).

Self-Determined Motivation. The 15-item Basic Psychological Needs Scale (BPNS; Ryan and Deci, 2000) was adapted to the pickleball context to assess three dimensions: Autonomy Need (5 items; e.g., “I can freely express my opinions about pickleball”), Competence Need (5 items; e.g., “I feel confident in my pickleball skills”), and Relatedness Need (5 items; e.g., “I maintain harmonious relationships with other players”). All items were activity-specific to pickleball, consistent with SDT measurement practice indicating that context-specific assessment improves predictive validity (Hagger et al., 2006). Cronbach's α coefficients were 0.876, 0.887, and 0.889 for the three dimensions, respectively. Confirmatory Factor Analysis (CFA) supported the three-factor structure (χ^2^/df = 1.442, RMSEA = 0.024, CFI = 0.988; see Table 3), with all factor loadings > 0.7, CR ranging from 0.877 to 0.890, and Average Variance Extracted (AVE) ranging from 0.588 to 0.618. In the structural model, the three dimensions were modeled as distinct exogenous variables to examine their differentiated effects on leisure specialization.

Leisure specialization was measured using the 19-item scale developed by Scott and Shafer (2001), comprising three sub-dimensions: cognitive mastery, behavioral involvement, and affective commitment. Responses were rated on a five-point Likert scale ranging from 1 (strongly disagree) to 5 (strongly agree). To reduce model complexity, minimize measurement error associated with individual items, and improve the stability of parameter estimates, a dimension-level item parceling strategy was adopted for the leisure specialization construct (Little et al., 2002). Specifically, the average score of all items within each sub-dimension was computed as a single observed indicator for the latent variable of leisure specialization, yielding three indicators corresponding to the three theoretical sub-dimensions. This approach is appropriate for multidimensional constructs with clearly delineated theoretical sub-dimensions, and has been widely used in structural equation modeling research within the sport and leisure literature (Little et al., 2002).

Continuance Intention was assessed using a four-item scale adapted from Wang et al. (2023) to measure participants' intention to continue playing pickleball (e.g., “I intend to continue playing pickleball in the future”). The Cronbach's α for this scale was 0.914.

### 4.3 Data analysis

Analyses were conducted in SPSS 24.0 and Amos 23.0, following the two-step approach recommended by Anderson and Gerbing (1988).

Measurement Model. Confirmatory Factor Analysis (CFA) was first conducted to evaluate the construct validity and reliability of the measurement scales. Model fit was assessed using multiple standard indices: the Chi-square/degrees of freedom ratio (χ^2^/df), Goodness-of-Fit Index (GFI), Adjusted Goodness-of-Fit Index (AGFI), Comparative Fit Index (CFI), Tucker-Lewis Index (TLI), and Root Mean Square Error of Approximation (RMSEA). Convergent validity was evaluated based on standardized factor loadings, Composite Reliability (CR), and Average Variance Extracted (AVE). Discriminant validity was assessed using the Fornell-Larcker criterion, and internal consistency was measured via Cronbach's α.

Structural Model. Following confirmation of an acceptable measurement model fit, Structural Equation Modeling (SEM) was employed to test the hypothesized direct relationships between variables. The mediating effect of leisure specialization was examined using the bias-corrected percentile Bootstrap method with 5,000 resamples, with effects considered statistically significant when the 95% bias-corrected confidence interval excluded zero (Preacher and Hayes, 2008).

Prior to conducting multi-group analysis of the moderating effect of gender, a series of measurement invariance tests were conducted following standard procedures (Cheung and Rensvold, 2002). Four nested models were tested sequentially: (1) configural invariance (the baseline model with identical factor structure across groups, no cross-group equality constraints), (2) metric invariance (equality of factor loadings across male and female groups), (3) scalar invariance (equality of item intercepts across groups), and (4) residual invariance (equality of item error variances across groups). Model fit was compared across nested models, with ΔCFI < 0.01 adopted as the criterion for accepting invariance, as this index is less sensitive to large sample sizes than the traditional Δχ^2^ test (Cheung and Rensvold, 2002). Establishment of scalar invariance was treated as a prerequisite for meaningful comparison of structural path coefficients across gender groups.

Once scalar invariance was confirmed, multi-group structural equation modeling was conducted to examine the moderating role of gender in the proposed motivational pathways.

### 4.4 Common method bias

Given the cross-sectional and self-reported nature of the data, the potential for common method bias (CMB) was assessed using Harman's single-factor test (Podsakoff et al., 2003). An unrotated exploratory factor analysis revealed that the first factor accounted for 34.94% of the total variance (see Table 4), below the recommended 40% threshold, suggesting that CMB is unlikely to pose a serious threat to the validity of the findings.

**Table 4:** Total variance explained for common method bias.

Component	Initial eigenvalues			Extraction sums of squared loadings			Rotation sums of squared loadings		
	**Total**	**% of variance**	**Cumulative %**	**Total**	**% of variance**	**Cumulative %**	**Total**	**% of variance**	**Cumulative %**
1	23.057	34.935	34.935	23.057	34.935	34.935	6.066	9.191	9.191
2	3.511	5.32	40.256	3.511	5.32	40.256	4.083	6.187	15.378
3	3.008	4.558	44.814	3.008	4.558	44.814	3.582	5.427	20.805
4	2.204	3.339	48.153	2.204	3.339	48.153	3.545	5.372	26.176
5	1.868	2.83	50.983	1.868	2.83	50.983	3.373	5.111	31.287
6	1.705	2.583	53.566	1.705	2.583	53.566	3.358	5.089	36.376
7	1.545	2.342	55.908	1.545	2.342	55.908	3.241	4.911	41.286
8	1.502	2.276	58.184	1.502	2.276	58.184	3.138	4.755	46.041
9	1.409	2.135	60.319	1.409	2.135	60.319	2.941	4.457	50.498
10	1.339	2.029	62.348	1.339	2.029	62.348	2.731	4.138	54.636
11	1.238	1.876	64.224	1.238	1.876	64.224	2.571	3.896	58.532
12	1.165	1.765	65.989	1.165	1.765	65.989	2.486	3.767	62.299
13	1.121	1.699	67.688	1.121	1.699	67.688	2.434	3.688	65.987
14	1.091	1.653	69.341	1.091	1.653	69.341	2.214	3.355	69.341

## 5 Results

### 5.1 Descriptive statistics and sample characteristics

Descriptive statistics for all study variables are shown in Table 2. The sample was predominantly composed of young to middle-aged adults, with the 20–49 age bracket accounting for 85.3% of respondents.

### 5.2 Measurement model evaluation

#### 5.2.1 Confirmatory factor analysis (CFA)

CFA was performed on the 789 valid responses. The measurement model demonstrated an excellent fit to the data (see Table 5 for detailed fit indices): χ^2^/df = 1.442, GFI = 0.957, AGFI = 0.948, RMSEA = 0.024, CFI = 0.988, TLI = 0.986 (Hu and Bentler, 1999).

**Table 5:** Measurement model fit indices.

**Fit index**	Criterion	Value	Evaluation
**Absolute fit indices**			
CMIN/DF	< 3	1.442	Excellent
GFI	>0.8	0.957	Excellent
AGFI	>0.8	0.948	Excellent
RMSEA	< 0.08	0.024	Excellent
**Incremental fit indices**			
NFI	>0.8	0.961	Excellent
IFI	>0.8	0.988	Excellent
TLI	>0.8	0.986	Excellent
CFI	>0.8	0.988	Excellent
**Parsimonious fit indices**			
PNFI	>0.5	0.848	Excellent
PCFI	>0.5	0.872	Excellent

All standardized factor loadings for the latent variables exceeded the recommended threshold of 0.70. As shown in Table 3, the Composite Reliability (CR) for all constructs ranged from 0.803 to 0.899, exceeding the 0.60 criterion, and the Average Variance Extracted (AVE) values ranged from 0.577 to 0.690, surpassing the 0.50 cutoff (Fornell and Larcker, 1981), jointly confirming adequate convergent validity. Discriminant validity was likewise established, as the square root of the AVE for each construct exceeded its correlations with all other constructs.

#### 5.2.2 Reliability analysis

Internal consistency was high for all scales. As presented in Table 6, Cronbach's α coefficients ranged from 0.876 to 0.914, exceeding the 0.70 benchmark (Nunnally, 1978). The test-retest reliability (*n* = 30, 4-week interval) also exceeded the 0.80 standard, confirming adequate temporal stability.

**Table 6:** Scale reliability analysis (*N* = 789).

**Construct**	Item	CITC	Cronbach's α if item deleted	Cronbach's α	Split-half reliability	Test-retest reliability (4 weeks)
Relatedness	REL1	0.800	0.848	0.889	0.872	0.851
	REL2	0.716	0.867			
	REL3	0.700	0.871			
	REL4	0.728	0.865			
	REL5	0.705	0.870			
Competence	COM6	0.795	0.847	0.887	0.865	0.842
	COM7	0.726	0.863			
	COM8	0.725	0.863			
	COM9	0.710	0.867			
	COM10	0.685	0.872			
Autonomy	FRE11	0.762	0.836	0.876	0.858	0.832
	FRE12	0.696	0.852			
	FRE13	0.685	0.854			
	FRE14	0.707	0.849			
	FRE15	0.681	0.855			
Cognitive mastery	COG1	0.740	0.811	0.858	0.832	0.815
	COG2	0.644	0.836			
	COG3	0.667	0.831			
	COG4	0.665	0.831			
	COG5	0.656	0.834			
Behavioral involvement	ACT6	0.758	0.826	0.870	0.845	0.823
	ACT7	0.689	0.844			
	ACT8	0.672	0.848			
	ACT9	0.686	0.844			
	ACT10	0.670	0.848			
Affective commitment	EMO11	0.761	0.899	0.914	0.901	0.882
	EMO12	0.694	0.904			
	EMO13	0.679	0.905			
	EMO14	0.690	0.904			
	EMO15	0.697	0.904			
	EMO16	0.700	0.904			
	EMO17	0.683	0.905			
	EMO18	0.690	0.904			
	EMO19	0.695	0.904			
Continuance intention	MPI1	0.819	0.850	0.897	0.885	0.861
	MPI2	0.785	0.862			
	MPI3	0.727	0.884			
	MPI4	0.764	0.870			

### 5.3 Structural model and hypothesis testing

#### 5.3.1 Model fit

The structural model exhibited a good fit to the data: χ^2^/df = 1.594, GFI = 0.952, AGFI = 0.942, RMSEA = 0.027, CFI = 0.983, TLI = 0.981, satisfying the recommended criteria (Hu and Bentler, 1999).

#### 5.3.2 Path coefficients

Standardized path coefficients and hypothesis testing results are summarized in Table 7 and illustrated in Figure 2. All three dimensions of self-determined motivation had significant positive effects on leisure specialization: competence showed the strongest effect (β = 0.285, *p* < 0.001), followed by relatedness (β = 0.202, *p* < 0.001) and autonomy (β = 0.179, *p* < 0.001), supporting H1–H3.

**Figure 2:**
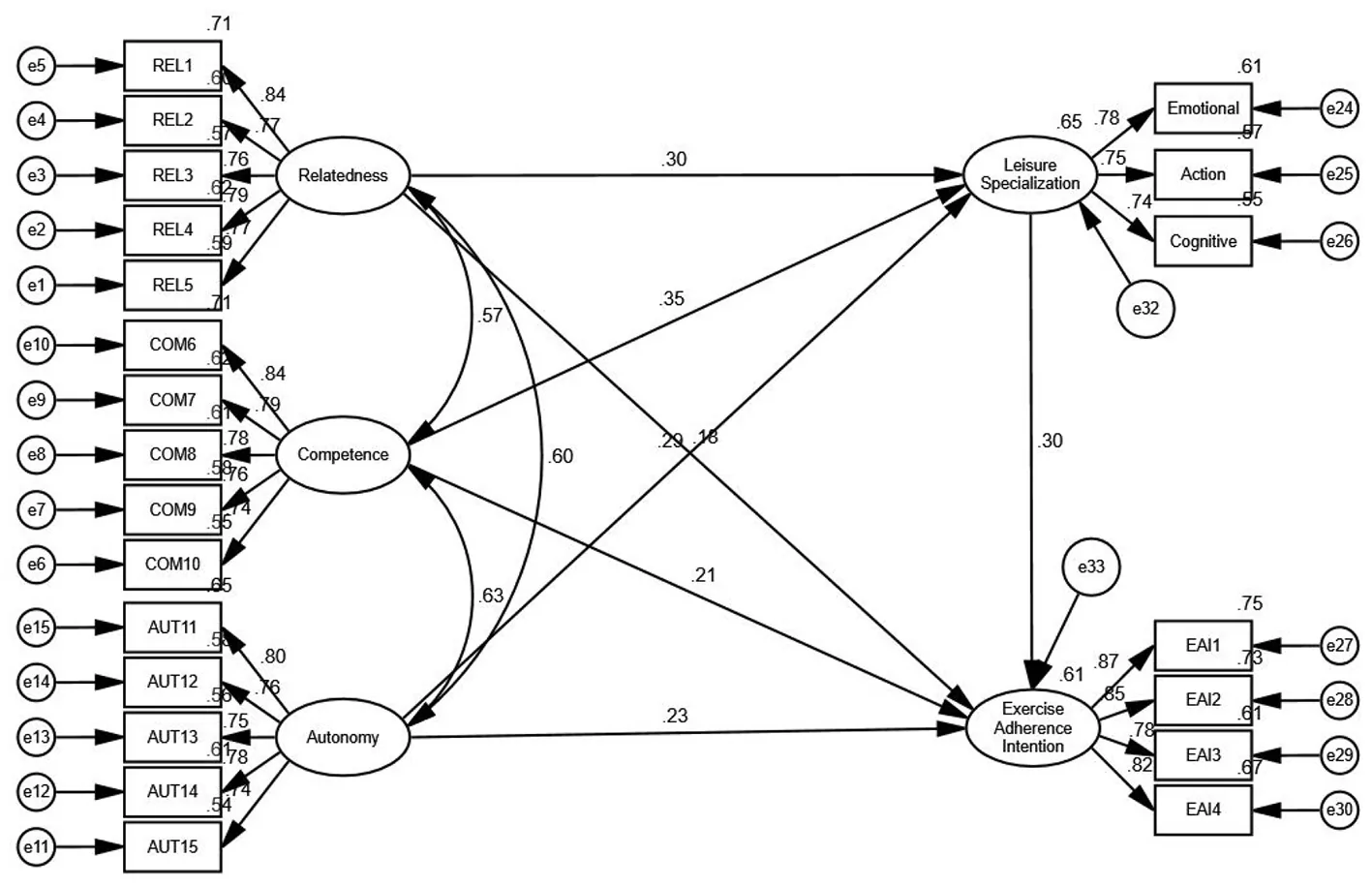
Structural equation model testing the hypothesized relationships.

**Table 7:** Structural equation model path coefficients and significance (*N* = 789).

Path	Std. estimate (β)	Unstd. estimate	**S.E**.	**C.R**.	***p***	Hypothesis
Relatedness → Leisure specialization	0.202	0.153	0.031	4.866	^***^	H3
Competence → Leisure specialization	0.285	0.225	0.034	6.691	^***^	H2
Autonomy → Leisure specialization	0.179	0.146	0.038	3.871	^***^	H1
Leisure specialization → Continuance intention	0.599	0.907	0.087	10.468	^***^	H4

^*^p < 0.05, ^**^p < 0.01, ^***^p < 0.001

Leisure specialization in turn had a significant positive effect on continuance intention (β = 0.599, *p* < 0.001), supporting H4.

#### 5.3.3 Mediation analysis

Bootstrap mediation analysis (Table 8) showed that leisure specialization significantly partially mediated the relationship between each need dimension and continuance intention. All 95% bias-corrected confidence intervals for indirect effects excluded zero, confirming significant mediation. The indirect effects were significant for relatedness (indirect effect = 0.030, 95% CI [0.005, 0.074]), competence (indirect effect = 0.042, 95% CI [0.007, 0.092]), and autonomy (indirect effect = 0.025, 95% CI [0.003, 0.065]). The proportion of the total effect mediated was 20.13% for relatedness, 19.35% for competence, and 15.24% for autonomy. These findings support H5.

**Table 8:** Mediation effect analysis results (*N* = 789; Bootstrap = 5,000).

Path	Effect type	Estimate	95% CI lower	95% CI upper	*p*
Relatedness → Leisure Spec. → Cont. Int.	Direct effect	0.120	0.024	0.211	0.015
	Indirect effect	0.030	0.005	0.074	0.018
	Total effect	0.149	0.053	0.237	0.002
Competence → Leisure Spec. → Cont. Int.	Direct effect	0.175	0.075	0.275	0.001
	Indirect effect	0.042	0.007	0.092	0.020
	Total effect	0.217	0.120	0.309	0.000
Autonomy → Leisure Spec. → Cont. Int.	Direct effect	0.140	0.035	0.239	0.011
	Indirect effect	0.025	0.003	0.065	0.022
	Total effect	0.164	0.059	0.261	0.004

^*^p < 0.05, ^**^p < 0.01, ^***^p < 0.001. Indirect effects are significant when the 95% bias-corrected confidence interval does not contain zero.

### 5.4 Multi-group analysis: the moderating role of gender (H6)

Prior to multi-group analysis, measurement invariance across gender groups was tested following the procedure described in the Method section. The configural invariance model demonstrated acceptable fit (χ^2^/df = 1.52, CFI = 0.984, TLI = 0.982, RMSEA = 0.026). Metric invariance was supported with ΔCFI = 0.003, and scalar invariance was supported with ΔCFI = 0.005, both meeting the < 0.01 invariance criterion (Cheung and Rensvold, 2002). Strong measurement invariance was thus established, warranting subsequent cross-group comparison of structural paths.

Multi-group analysis revealed that while the overall model difference between genders was significant [Δχ^2^_(23)_ = 28.96, *p* = 0.018], critical ratios for all individual path differences were below the 1.96 threshold (see Table 9). Specifically, the paths for Competence → Leisure Specialization (CR = 1.79, *p* = 0.073), Autonomy → Leisure Specialization (CR = 0.78, *p* = 0.435), and Relatedness → Leisure Specialization (CR = 0.42, *p* = 0.674) did not differ significantly across groups. H6 was therefore not supported: despite the marginally significant overall chi-square difference, which is likely inflated by the large total sample size (Cheung and Rensvold, 2002), none of the core motivational pathways from psychological needs to behavioral control differed significantly across genders. Sensitivity analyses using male subsamples matched to female sample size (Tables 10a, 10b) yielded consistent results, confirming the findings were not biased by sample size imbalance.

**Table 9:** Multi-group analysis by gender.

Path	Males (***n*** = **507)**					Females (***n*** = **282)**				
	***B***	β	**S.E**.	**C.R**.	***P***	***B***	β	**S.E**.	**C.R**.	***P***
Relatedness → Leisure specialization	0.144	0.197	0.038	3.741	^***^	0.169	0.209	0.055	3.063	0.002
Competence → Leisure specialization	0.192	0.261	0.038	5.010	^***^	0.319	0.352	0.068	4.670	^***^
Autonomy → Leisure specialization	0.121	0.156	0.046	2.659	0.008	0.179	0.200	0.069	2.603	0.009
Leisure spec. → Cont. intention	0.962	0.658	0.112	8.626	^***^	0.841	0.525	0.138	6.077	^***^

^*^p < 0.05, ^**^p < 0.01, ^***^p < 0.001. The overall model difference is significant (Δχ^2^), but no single path differs significantly across groups (|CR| < 1.96).

**Table 10A:** Sensitivity analysis 1: subsampling MGA results (male *n* = 282 vs. female *n* = 282).

**Path**	Males (***n*** = **282, matched subsample)**					Females (***n*** = **282)**				
	***B***	β	**S.E**.	**C.R**.	***P***	***B***	β	**S.E**.	**C.R**.	***P***
Relatedness → Leisure specialization	0.191	0.242	0.042	4.497	^***^	0.191	0.212	0.042	4.497	^***^
Competence → Leisure specialization	0.265	0.289	0.049	5.382	^***^	0.265	0.272	0.049	5.382	^***^
Autonomy → Leisure specialization	0.192	0.212	0.055	3.462	^***^	0.192	0.196	0.055	3.462	^***^
Leisure specialization → Continuance intention	1.053	0.698	0.107	9.880	^***^	1.053	0.649	0.107	9.880	^***^

^*^p < 0.05, ^**^p < 0.01, ^***^p < 0.001. Results are based on randomly matched male subsamples (n = 282) to balance sample size with the female group; non-significant path differences are consistent with full-sample MGA results.

**Table 10B:** Sensitivity analysis 2: subsampling MGA results (male *n* = 282 vs. female *n* = 282, repeated sampling).

**Path**	Males (***n*** = **282**, matched subsample)					Females (***n*** = 282)				
	***B***	β	**S.E**.	**C.R**.	***P***	***B***	β	**S.E**.	**C.R**.	***P***
Relatedness → Leisure specialization	0.187	0.231	0.044	4.248	^***^	0.187	0.202	0.044	4.248	^***^
Competence → Leisure specialization	0.212	0.245	0.047	4.536	^***^	0.212	0.212	0.047	4.536	^***^
Autonomy → Leisure specialization	0.210	0.231	0.055	3.810	^***^	0.210	0.209	0.055	3.810	^***^
Leisure specialization → Continuance intention	1.035	0.666	0.114	9.109	^***^	1.035	0.627	0.114	9.109	^***^

^*^p < 0.05, ^**^p < 0.01, ^***^p < 0.001. Results are based on randomly matched male subsamples (n = 282) to balance sample size with the female group; non-significant path differences are consistent with full-sample MGA results.

## 6 Discussion

Findings are interpreted with reference to the tournament player sample described in Section 4.1; generalization to recreational populations should be made with caution.

### 6.1 Empirical support for the integrated SDT-TPB framework

#### 6.1.1 Validating leisure specialization as PBC

This study provides empirical evidence for an integrated SDT-TPB pathway in a low-skill-threshold sport context. Leisure specialization, operationalized as perceived behavioral control (PBC), functions as a crucial bridging mechanism in the “Psychological Needs → Behavioral Control → Continuance Intention” chain. The verified pathways are visually synthesized in Figure 3, which illustrates the complete chain from psychological needs (autonomy, competence, relatedness) through leisure specialization (as behavioral control) to continuance intention, alongside the non-significant moderating role of gender. The theoretical alignment between leisure specialization and PBC (Table 1) is empirically substantiated by three strands of evidence.

**Figure 3:**
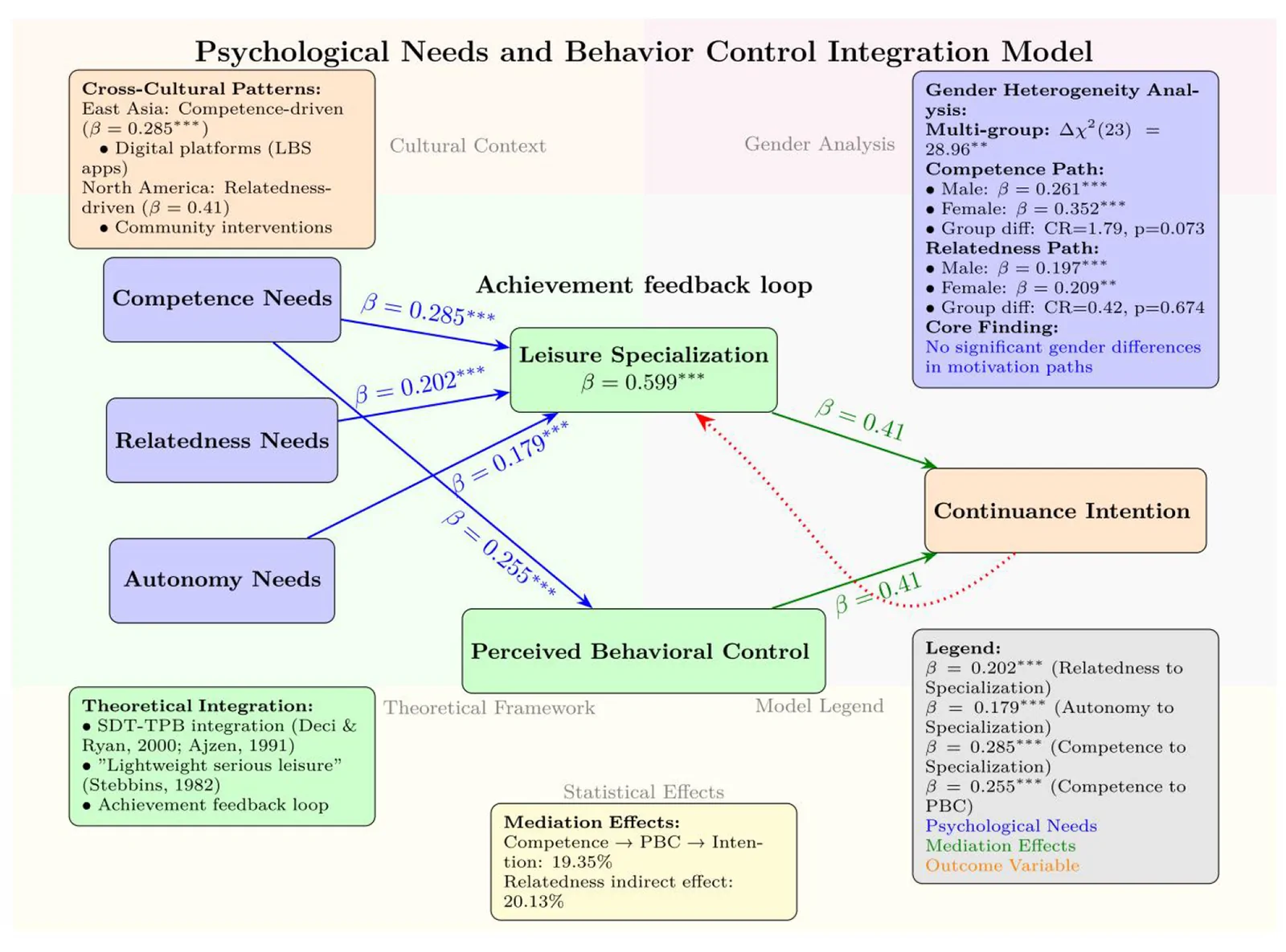
Integrated framework of psychological needs and behavioral control mechanisms. *p < 0.05, **p < 0.01, ***p < 0.001.

First, the results support the theoretical proposition that basic psychological need satisfaction translates into behavioral intention via behavioral control mechanisms (Vallerand, 1997). Significant partial mediation for all three need dimensions—autonomy (15.24%), competence (19.35%), relatedness (20.13%)—corroborates this mechanism in low-skill-threshold sports, with all Bootstrap 95% confidence intervals excluding zero (Table 8). Notably, relatedness need exhibited the highest mediation proportion (20.13%), suggesting that social connection, when internalized through affective commitment to the activity, plays a particularly influential role in translating psychological needs into sustained participation. Leisure specialization not only reflects the behavioral control component of TPB (Ajzen, 1991) but also represents a behavioral expression of internalized psychological needs (Lee and Ewert, 2018), thereby bridging the motivational (SDT) and volitional (TPB) phases of behavioral regulation.

Second, the path coefficients directly validate the three dimensions aligned in Table 1. The significant predictive effect of competence need on specialization confirms that skill development—central to the cognitive mastery dimension—directly enhances perceived behavioral control. Likewise, the mediation effect of relatedness through specialization (indirect effect = 0.030, *p* < 0.05) demonstrates that social connections are internalized through affective commitment, reinforcing perceived ease of participation. Collectively, these findings empirically substantiate the theoretical correspondence between leisure specialization dimensions (behavioral involvement, cognitive mastery, affective commitment) and the core connotations of PBC (behavioral execution control, perceived capability, affective commitment value) as outlined in Table 1. This operationalization responds to calls in the literature for more domain-specific indicators of PBC (Hagger et al., 2006), capturing concrete behavioral manifestations of control—resource investment, knowledge accumulation, and emotional attachment—rather than relying solely on general perceptions. Compared with general, self-reported PBC scales that capture broad perceived control, this behavioral operationalization provides more concrete, actionable indicators for practitioners. This approach is particularly relevant in low-skill-threshold, socially oriented sports like pickleball, offering clear targets for intervention design to promote sustained participation.

Third, cross-cultural contrasts help delineate the boundary conditions of the proposed framework. The present young and middle-aged Chinese sample contrasts sharply with Western pickleball studies that have primarily focused on older adults (mean age 68.2 years in Casper and Jeon, 2018; participants aged ≥65 years accounting for 74% in Heo et al., 2018). The finding that competence need was the strongest driver of specialization (β = 0.285; see Table 7) in the Chinese sample, whereas relatedness was dominant in Western older-adult samples (Heo et al., 2018), suggests that cultural and generational contexts shape the relative importance of different psychological needs and, consequently, the manifestation of PBC through leisure specialization. The observed divergence may stem from both cultural and age-related factors. Extant Western pickleball research has predominantly sampled older adults, whereas the present sample is composed largely of young and middle-aged adults. Future research controlling for age groups is therefore needed to disentangle the independent effects of culture and generation. This divergence underscores the importance of considering both generational and cultural contexts when interpreting motivational mechanisms in sport participation. We note that the present design cannot fully disentangle age/cohort effects from cultural effects, and the results highlight the need for caution when generalizing findings from Western older-adult populations to East Asian young and middle-aged populations.

#### 6.1.2 Competence need as the primary driver

Competence emerged as the strongest direct predictor of leisure specialization (β = 0.285, *p* < 0.001), warranting careful interpretation. As shown in Figure 3, this path had the largest coefficient among the three needs, reinforcing competence as the primary driver of specialization in this sample of young and middle-aged Chinese tournament players. This pattern is first attributable to the distinctive characteristics of pickleball as a low-barrier sport: its simplified rules, short paddles, and lightweight balls create an accelerated feedback cycle for skill development and achievement. Unlike traditional racquet sports, where mastery may require years of practice, pickleball allows beginners to reach recreational competitive levels relatively quickly (International Federation of Pickleball, 2023). This rapid, visible competence feedback is likely to reinforce continued time, energy and skill investment, accelerating the process of leisure specialization.

Furthermore, the cultural and demographic context of the sample may amplify the salience of competence need. The participants were predominantly young and middle-aged adults at a life stage marked by achievement orientation, and Chinese social contexts further tie educational, professional and leisure success to skill demonstration and mastery (Chen and Tsai, 2020; Zhen et al., 2011). In this context, opportunities to develop and display competence in a new, accessible sport may be particularly attractive to participants. This helps explain why competence, rather than relatedness, emerged as the dominant predictor in the present sample—a pattern that challenges the widely held assumption, derived primarily from Western older-adult samples (Heo et al., 2018; Casper and Jeon, 2018), that pickleball participation is universally driven primarily by social connection.

Notably, in comparison to the strong effect of competence, autonomy exhibited a significant but notably smaller positive effect on leisure specialization (β = 0.179, *p* < 0.001), potentially reflecting context-dependent expression of autonomy in East Asian cultural settings (Berry, 1997). Within this context, autonomy need satisfaction may manifest more as “autonomy within structure” (Tang et al., 2021) rather than pure independent decision-making, suggesting cultural adaptability shapes how basic psychological needs are expressed and prioritized in sport settings.

#### 6.1.3 Relatedness as the mediating mechanism

The finding that relatedness need exhibited the highest mediation proportion (20.13%) through leisure specialization (see Figure 3 for the overall mediation framework) underscores the importance of social mechanisms in translating psychological needs into sustained participation. Theoretically, this may be understood as the internalization of emotional bonds through social interaction (Kipp and Weiss, 2013). Pickleball's predominantly mixed-gender format creates opportunities for cross-generational and cross-gender social connections, which may facilitate specialization by strengthening emotional bonds and a sense of belonging to the activity.

The pronounced mediation proportion for relatedness need also has important implications for understanding persistence mechanisms. Whereas, competence need may initiate participation by providing the challenge and skill development that attracts participants, relatedness need sustains participation by embedding it within a social context that provides meaning, support, and community. This sequential pattern suggests that effective retention strategies must address both the initial attraction (competence) and the sustaining relationships (relatedness) that characterize long-term participation.

#### 6.1.4 Cross-gender universality

Turning to gender differences in the motivational pathways, the non-significant moderation of gender on the core motivation-specialization paths is particularly noteworthy. As visually summarized in Figure 3, despite a marginally significant overall model chi-square difference (driven primarily by large sample size), the core paths from psychological needs to behavioral control (leisure specialization), and from leisure specialization to continuance intention, are largely invariant across genders: none of the individual path critical ratios meet the 1.96 threshold for statistical significance (competence: CR = 1.79, *p* = 0.073; autonomy: CR = 0.78, *p* = 0.435; relatedness: CR = 0.42, *p* = 0.674).

Contrary to traditional gender role stereotypes that posit inherent, stable motivational differences between men and women in competitive sport, this pattern aligns with our hypothesis, providing preliminary correlational evidence consistent with the possibility that low-skill-threshold, mixed-gender sport designs may attenuate traditional gender-role constraints on sport motivation. This finding adds to a growing body of empirical evidence challenging universal, essentialist assumptions about inherent gender differences in sport motivation. For instance, Manzano-Sánchez (2023) found no gender differences in motivational profiles among 752 students, consistent with similar findings in other low-barrier sport contexts (Cerezuela et al., 2023). Similarly, Li et al. (2025) reported that while gender moderated specific peripheral pathways in an integrated BPN-TPB model, the core motivational mechanisms operated consistently across genders.

Nevertheless, the modest overall model difference suggests that gender may influence persistence through secondary pathways not examined in the present analysis. One promising direction for future research with larger samples is to test whether leisure specialization dimensions—particularly behavioral involvement and affective commitment—exert differential effects on continuance intention across genders: for example, behavioral investment may predict continuance more strongly for males, while affective commitment may be more influential for females. Such dimension-level moderation analyses were not powered in the present study, but represent an important extension of the current findings.

### 6.2 Core findings based on generational and cultural differences

Consistent with the sample characteristics noted above, Chinese pickleball participants in this study were primarily young and middle-aged adults, with competence need as the core driver of persistence. North America exhibits a dual-track pattern: a broad age distribution where younger adults (18–44 years) constitute 70.5% of participants and are the primary growth driver (mean age decreasing to 35 years), while seniors (65+) remain a vital core segment, comprising 20.8% (SFIA Pickleheads, 2023). Understanding these generational and cultural disparities is essential for designing culturally adapted strategies optimizing the “Psychological Needs → Leisure Specialization → Continuance Intention” chain.

In the Chinese sample (85.3% young/middle-aged adults), this demographic concentration makes competence the primary driver of leisure specialization, reflecting participants' desire to build sport identity through skill mastery. The achievement feedback loop directly reinforces perceived behavioral control (Ryu et al., 2020). In North America (dual-track—young growth and senior core), young adults (18–44 years, 70.5%; fastest growth in 18–24) are significantly influenced by competitive-recreational formats. Seniors (65+, 20.8%) display a pronounced reliance on relatedness need for persistence, requiring community health-embedded strategies. A key cross-cultural commonality is the universal importance of relatedness need mediation (20.13%), facilitated by pickleball's mixed formats serving diverse generational needs.

Successful cultural adaptation requires focusing promotion on the dominant participant group using differentiated vehicles, tightly aligned with the study's core findings. For the Chinese young/middle-aged context, strategies that provide clear skill progression and visible achievement feedback may effectively address the strongest driver identified—competence need—by reinforcing perceived behavioral control through recognizable progress. Concurrently, interventions that facilitate social matching and organized group activities could address the high mediated effect of relatedness need by reducing barriers to social connection, accelerating belongingness internalization and consolidating behavioral control.

### 6.3 Boundaries of applicability

This study is based on a sample of Chinese competitive pickleball participants, and the findings are most directly applicable to this group. The cross-cultural comparisons discussed (e.g., contrasts with Western research) are intended to generate hypotheses for future cross-cultural research rather than to establish universal conclusions. Generalization of the findings to other cultural contexts (e.g., other East Asian countries, Western countries) or other participant groups (e.g., recreational participants, novices) should be made with caution and requires further empirical validation.

### 6.4 Theoretical contributions

Beyond empirically validating the leisure-specialization-as-PBC framework, this study makes three key theoretical contributions to the sport and leisure motivation literature, each addressing identified gaps in prior research.

First, it reveals differentiated mediation patterns across the three dimensions of basic psychological needs. Prior integrated SDT-TPB studies have mostly treated need satisfaction as a unidimensional aggregate construct (Gucciardi and Jackson, 2015; Li et al., 2025), without testing the relative strength of different need dimensions in shaping domain-specific behavioral control. The finding that relatedness exhibits the highest proportion of mediated effect (20.13%), while autonomy shows the lowest (15.24%), demonstrates that not all psychological needs are equally conducive to the internalization of perceived behavioral control through leisure specialization. This nuance extends prior work by highlighting that social connection—via affective internalization—serves as a particularly robust pathway for consolidating domain-specific behavioral control.

Second, it extends the external validity of the integrated framework to an understudied demographic and cultural context. Existing pickleball motivation research has overwhelmingly focused on Western older-adult samples (Heo et al., 2018; Casper and Jeon, 2018; Ryu et al., 2020), with limited evidence from East Asian young and middle-aged populations who now represent the fastest-growing segment of pickleball participants globally. The notable contrast between our young and middle-aged Chinese sample and Western older-adult samples indicates that the relative salience of basic psychological needs is contingent on generational and cultural contexts. This finding cautions against the uncritical generalization of motivational models across populations, and underscores the importance of developing culturally and demographically situated models of sport participation persistence.

Third, it contributes to the ongoing debate on gender differences in sport motivation. Early sport motivation research often documented stable gender differences in motivational orientations (Chalabaev et al., 2013), while recent studies have increasingly challenged the universality of such differences, particularly in low-barrier, mixed-gender sport contexts (Manzano-Sánchez, 2023; Cerezuela et al., 2023; Li et al., 2025). The non-significant moderating effect of gender on the core motivational pathways aligns with this recent evidence, and further implies that sport-specific features—such as low skill thresholds and mixed-gender participation formats—can attenuate traditional gender-role constraints, providing theoretical support for a context-dependent rather than gender-essentialist account of sport motivation. Beyond pickleball, this contextualized PBC operationalization may also be applicable to other emerging low-threshold racket and social sports (e.g., padel, ultimate frisbee) with similar participation characteristics, offering a transferable framework for studying sustained participation in the growing category of accessible, socially oriented new sports.

### 6.5 Practical implications

The following practical implications are derived directly from the empirical results and should be interpreted in light of the study's sample characteristics (competitive tournament participants; see Section 4.1).

Addressing Competence Need (direct effect: β = 0.285, *p* < 0.001, the strongest). Practitioners may consider implementing structured skill progression systems (e.g., tiered certification or rating benchmarks) that provide visible performance feedback, thereby reinforcing the perceived behavioral control that sustains continuance intention.

Strengthening Relatedness Need (indirect effect proportion: 20.13%, the highest). Organizers may promote mixed-doubles formats and organized social play events that expand participants' social networks. Integrating pickleball into existing community or workplace social infrastructures may further enhance the affective commitment that facilitates specialization.

Supporting Autonomy Need (direct effect: β = 0.179, *p* < 0.001, significant but weaker). Ensuring flexible participation options (e.g., varied scheduling, choice of partners) and reducing practical barriers (e.g., court availability, equipment access) is sufficient to support autonomy without requiring substantial additional resources.

Interpreting Implications in Context. These implications are derived from tournament players with consistent participation habits (39.8% participating in ≥3 sessions per week), and are most directly applicable to structured, competitive play settings. When extending these strategies to recreational or novice groups, practitioners should recognize that motivational priorities may differ for new players who have not yet developed skill mastery or social ties to the sport; pilot testing and targeted needs assessment are encouraged before full-scale implementation. Cross-cultural application of these strategies also requires further empirical validation.

### 6.6 Limitations and future research

While this study systematically explored the mechanisms driving pickleball continuance participation via self-determined motivation, several limitations warrant attention in future research:

Sampling Bias and Representativeness: The tournament-based purposive sampling strategy may overestimate the strength of competence need as a driver of specialization, as competitive players are more likely to prioritize skill improvement than casual recreational players, novices, or older adult participants who play primarily for social connection. The current findings also cannot capture motivational differences across participation stages (e.g., initial trial vs. long-term engagement). As noted in Section 6.5, the present implications are most applicable to high-involvement groups, and future research should use stratified sampling across community recreational players, novices, and senior groups to map motivational evolution across the participant lifecycle.

Cross-Sectional Design Limitation: The cross-sectional design only captures static data at a single time point, which cannot reveal the dynamic evolution of motivation, specialization, and intention over time, nor can it establish strict causal relationships between variables. While the structural equation modeling results are consistent with the proposed theoretical pathway, they do not verify causal direction or validate the underlying psychological mechanisms. The mediating effects of leisure specialization should be interpreted as statistical associations consistent with theoretical expectations, rather than confirmed causal chains. Future Direction: Adopt longitudinal designs combined with Ecological Momentary Assessment (EMA) via mobile apps for real-time behavioral data, enabling dynamic modeling of motivation-behavior interactions.

Limited Scope of Cultural Moderation: Cross-cultural comparison relied mainly on comparisons between the present Chinese sample and prior Western samples in the literature. Mechanisms may differ in broader cultural contexts. Future Direction: Expand to diverse cultural samples and explore regional economic levels and social capital as potential moderators to deepen understanding of cultural adaptability.

Unclear Critical Threshold of Specialization: While leisure specialization's mediating role is supported, its critical threshold for impacting continuance intention remains unknown. Future Direction: Utilize machine learning algorithms integrating skill proficiency (IFP standards), objective physiological metrics, and subjective psychological indicators to model non-linear relationships and detect potential specialization thresholds.

Unexplored Age/Lifecycle Moderation: The sample was predominantly young/middle-aged, emphasizing competence-driven mechanisms. Motivational pathways likely shift across the lifespan. Future Direction: Conduct cross-generational longitudinal studies comparing motivational pathways across age groups to design age-adapted intervention strategies.

## 7 Conclusion

This study examined the psychological mechanisms driving sustained participation in pickleball—a rapidly growing low-barrier racket sport—among young and middle-aged Chinese tournament participants. Drawing on an integrated SDT-TPB framework, we tested a model in which the three basic psychological needs (autonomy, competence, relatedness) influence continuance intention via the mediating role of leisure specialization, with gender as a potential moderator.

Analysis of cross-sectional survey data from 789 tournament participants was consistent with the hypothesized model, demonstrating that leisure specialization functions as a robust partial mediator linking need satisfaction to continuance intention. The three need dimensions exhibited distinct patterns of influence: competence emerged as the strongest direct predictor of specialization, while relatedness explained the largest proportion of the indirect effect on continuance intention. Notably, no significant gender differences were found in the core motivational pathways, providing preliminary evidence that low-barrier, socially oriented sports may attenuate traditional gender gaps in sport motivation.

Taken together, these findings advance understanding of sport continuance by extending the integrated SDT-TPB framework to a new cultural and demographic context, highlighting the central role of domain-specific behavioral investment in translating need satisfaction into long-term participation. The results offer actionable insights for sport managers and policymakers promoting lifelong racket sport participation, while pointing to key directions for future research on the dynamic longitudinal processes underlying sustained engagement. As pickleball and similar accessible recreational sports continue to grow globally, this study provides an evidence-based foundation for fostering long-term participation among adult populations.

## Data Availability

The raw data supporting the conclusions of this article will be made available by the authors, without undue reservation.
